# Influences of Diurnal Rhythms on Gut Microbiota and Clock Gene Expression in *Procambarus clarkii*

**DOI:** 10.3390/clockssleep8020029

**Published:** 2026-05-25

**Authors:** Lin Feng, Zhengyan Zhou, Yubo Ma, Yingying Zhao, Hua Wei, Xiaochen Zhu

**Affiliations:** 1 College of Life Science and Engineering, Shenyang University, Shenyang 110044, China; fl20010902@126.com (L.F.); zhouzhengyan@126.com (Z.Z.); 2 College of Animal Science and Veterinary Medicine, Shenyang Agricultural University, Shenyang 110866, China; yubo1932491347@163.com (Y.M.); zhaoyy@syau.edu.cn (Y.Z.); 3 College of Science and Engineering, Flinders University, Adelaide 5042, Australia

**Keywords:** diurnal rhythms, *Procambarus clarkii*, gut microbiota, clock genes, mRNA expression

## Abstract

This study investigated the diurnal dynamics of the gut microbiota and core clock gene expression in the red swamp crayfish, *Procambarus clarkii*. Samples were collected at four time points (06:00, 12:00, 18:00, and 24:00) over a 24 h period. Gut microbiota characteristics were analyzed using 16S rRNA gene sequencing, while quantitative real-time polymerase chain reaction (qPCR) was used to examine the expression patterns of core clock genes, including *Cycle* (*Cyc*), *Clock* (*Clk*), and *cryptochrome type 1* (*Cry1*), in the hepatopancreas and eyestalk. The results showed that Kruskal–Wallis tests for α-diversity indices (Shannon, Simpson, ACE, Chao1) and PERMANOVA for β-diversity (Bray–Curtis) of the gut microbiota revealed no significant diurnal variation across the four time points (all *p* > 0.05). Firmicutes, Proteobacteria, and Bacteroidota were the dominant phyla, with norank_o_RsaHf231, ZOR0006, and Bacteroides as the predominant genera. Although the overall microbial structure remained stable, several taxa, including *unclassified_c_Bacilli*, *unclassified_f_Caulobacteraceae*, *Gemmobacter*, *unclassified_f_Rhodobacteraceae*, *Allorhizobium–Neorhizobium–Pararhizobium–Rhizobium*, *Lactobacillus*, and *unclassified_f_Vibrionaceae* exhibited time-dependent fluctuations. In addition, the relative mRNA expression levels of *Cyc*, *Clk*, and *Cry1* in the hepatopancreas and eyestalk showed significant diurnal variation. This study reveals the diurnal dynamic characteristics of the gut microbiota and core clock gene expression in *P. clarkii*, providing a foundation for further investigation of diurnal regulatory mechanisms and physiological adaptations.

## 1. Introduction

Circadian rhythms refer to the predictable endogenous biological oscillations with an approximately 24 h period that enable organisms to synchronize physiological processes with daily environmental cycles [1]. These rhythms arise from long-term adaptation to periodic changes in external factors, such as the light cycle, and are manifested as circadian patterns in organism behavior and physiology. In aquatic animals, circadian rhythms are of great significance in regulating multiple biological processes, including immunity [2], metabolism [3], growth [4], and development [5]. Therefore, elucidating the regulatory basis of circadian rhythms in economically important aquatic animals can improve yields and optimize management strategies [6]. As a potent environmental driver, photoperiod (or the light–dark cycle) can shape the dynamic structure of the host gut microbiota. Previous studies on *Eriocheir sinensis* [7], *Palaemonetes sinensis* [8], and *Neocaridina denticulata* [9] have demonstrated that the composition and abundance of gut microbiota undergo diurnal changes in response to photoperiod or circadian rhythms. The gut microbiota plays essential roles in host digestion, energy absorption and utilization, immune regulation, and susceptibility to disease [10]. Therefore, investigating the diurnal variation in gut microbiota is critical for a comprehensive understanding of how biological rhythms influence host physiology and health.

At the molecular level, circadian rhythms are regulated by clock genes that constitute interconnected transcriptional–translational feedback loops. These clock genes exhibit approximately 24 h oscillations in expression and function as the core regulators of rhythmic physiological, biochemical, and behavioral processes [11]. In aquatic organisms, rhythmicity has been closely associated with the time-dependent expression of clock genes. For example, studies in *Homarus americanus* [12] and *Danio rerio* [13] have revealed pronounced time-dependent variations in clock gene expression.

*Procambarus clarkii* has become an important aquaculture species due to its strong adaptability, high fecundity, short growth cycle, and high market value [14]. Since gut microbiota critically influences nutrient metabolism, immunity, and disease resistance in crustaceans, deciphering its diurnal rhythmicity, which directly affects feed efficiency and health status under intensive culture conditions, is essential for optimizing feeding strategies and reducing production costs in crayfish aquaculture [15,16,17]. Despite the significant importance of this species in aquaculture, the impact of diurnal rhythms on its gut microbiota and rhythm-related gene expression remains poorly understood. In this study, we investigated the microbiota variation and the expression of three core clock genes on a daily basis. These findings could expand the chronobiology of crustaceans and provide a theoretical basis for enhancing crayfish aquaculture productivity through rhythm-based management.

## 2. Results

### 2.1. Gut Bacterial Sequencing Overview

Following quality filtering and denoising, a total of 1,292,661 high-quality reads were retained, with an average read length of 425 bp. Amplicon sequence variants (ASVs) were identified across all taxonomic ranks, yielding 2825 unique ASVs, including 20 at the phylum level, 374 at the genus level, and 503 at the species level.

**Figure 1:**
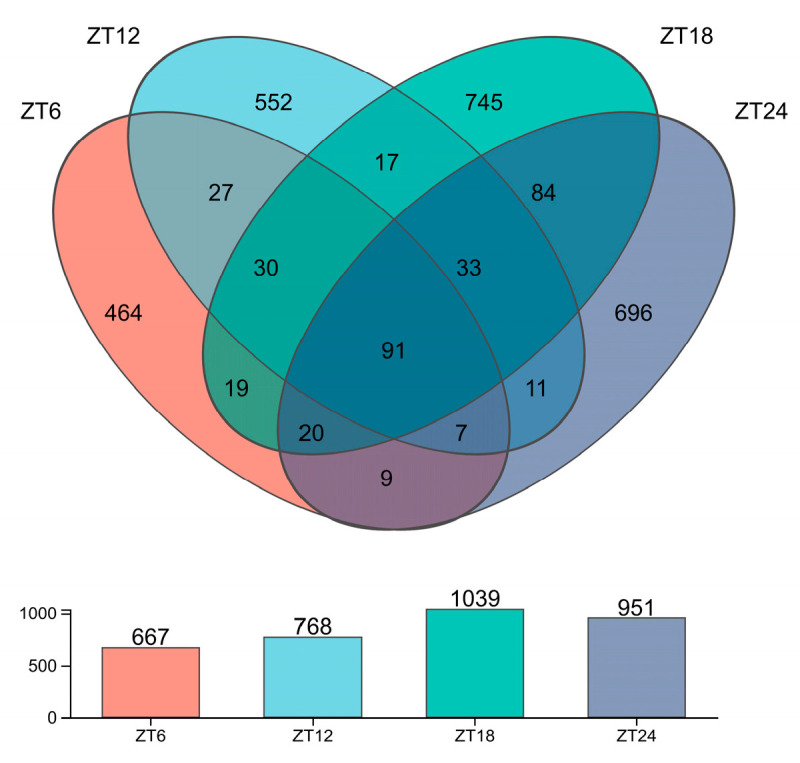
Venn diagram showing the shared and unique ASVs among the four time points.

As illustrated in the Venn diagram (Figure 1), only 91 gut microbial taxa were shared across the four time points, while each time point exhibited distinct, time-specific microbial taxa. Specifically, 464 taxa were exclusive to ZT6 (06:00), 552 to ZT12 (12:00), 745 to ZT18 (18:00), and 696 to ZT24 (24:00).

### 2.2. Diurnal Variation in Gut Bacterial Community Structure

**Figure 2:**
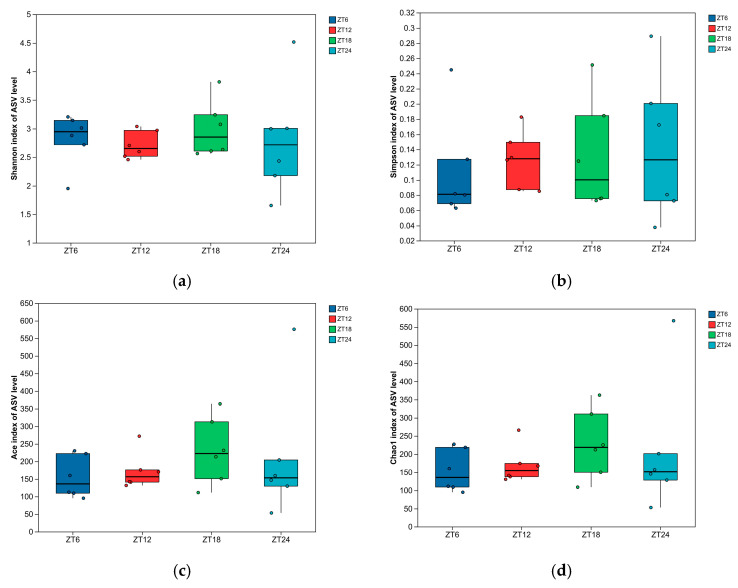
α-diversity indices of bacterial communities across four time points. (**a**) Shannon index; (**b**) Simpson index; (**c**) ACE index; (**d**) Chao1 index.

To evaluate changes in microbial community structure, α-diversity indices were determined at four sampling time points. As shown in Figure 2, Kruskal–Wallis tests revealed no statistically significant differences for the Shannon, Simpson, ACE, or Chao1 index (all *p* > 0.05).

**Figure 3:**
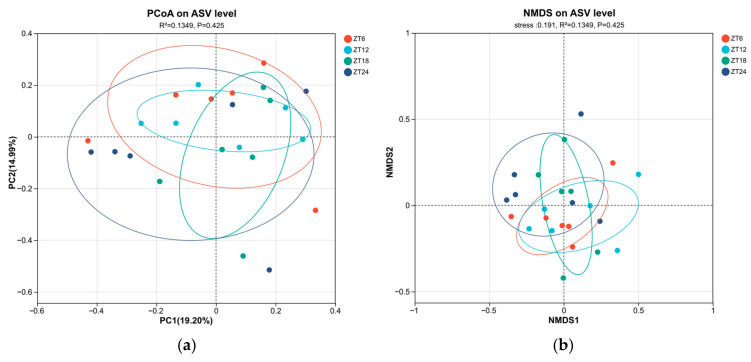
β-diversity analysis of bacterial communities across four daily time points. (**a**) Principal coordinate analysis (PCoA); (**b**) non-metric multidimensional scaling (NMDS) plot.

Principal coordinate analysis (PCoA) and non-metric multidimensional scaling (NMDS) were performed based on the Bray–Curtis dissimilarity matrix (Figure 3) to assess β-diversity. PERMANOVA for β-diversity revealed no significant separation among the gut microbial communities across the four time points (all *p* > 0.05).

### 2.3. Diurnal Variation in Gut Bacterial Taxonomic Abundance

**Figure 4:**
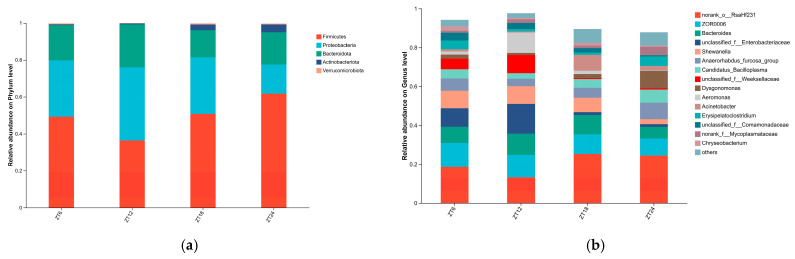
The relative abundance of ASVs across four time points. (**a**) Phylum level; (**b**) genera level.

The relative abundance of gut microbial taxa exhibited time-dependent fluctuations. The predominant bacterial phyla of crayfish were Firmicutes, Proteobacteria, Bacteroidota, and Actinobacteriota (Figure 4a). The predominant genera were *norank_o__RsaHf231*, *ZOR0006*, *Bacteroides*, *Shewanella*, and *unclassified_f__Enterobacteriaceae* (Figure 4b).

**Figure 5:**
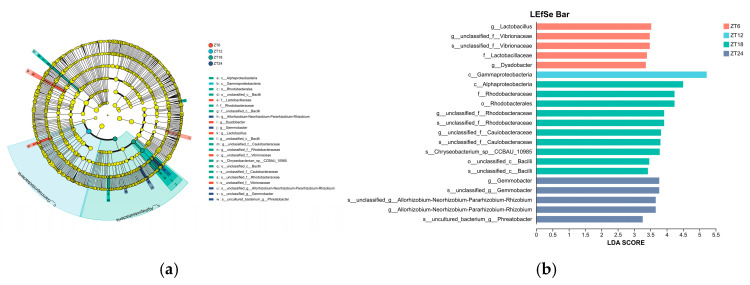
LEfSe analysis identifying the differential taxa across the four time points. (**a**) Cladogram showing the phylogenetic distribution of differential taxa; (**b**) LDA scores for significantly different taxa (LDA score > 2.0).

To identify the differential microbial taxa among the time points, LEfSe analysis was performed (Figure 5), and a total of 21 taxa were identified as differential taxa. Specifically, the genera Lactobacillus and Dyadobacter, and members of family Vibrionaceae, were significantly enriched at 06:00. The class Gammaproteobacteria showed higher abundance at 12:00. At 18:00, the class Alphaproteobacteria, members of the order Rhodobacteraceae, and Bacilli were significantly enriched. Members of the genera *Gemmobacter*, *Pheatobacter*, and *Allorhizobium–Neorhizobium–Pararhizobium–Rhizobium* showed higher abundance at 24:00.

**Figure 6:**
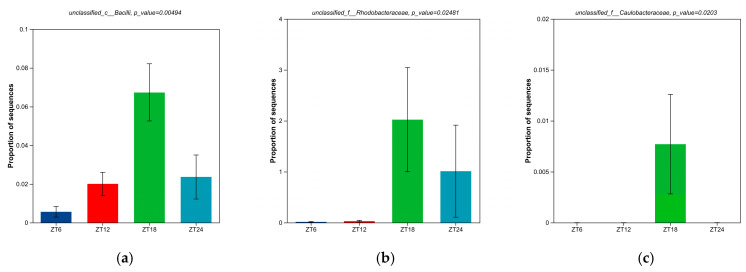

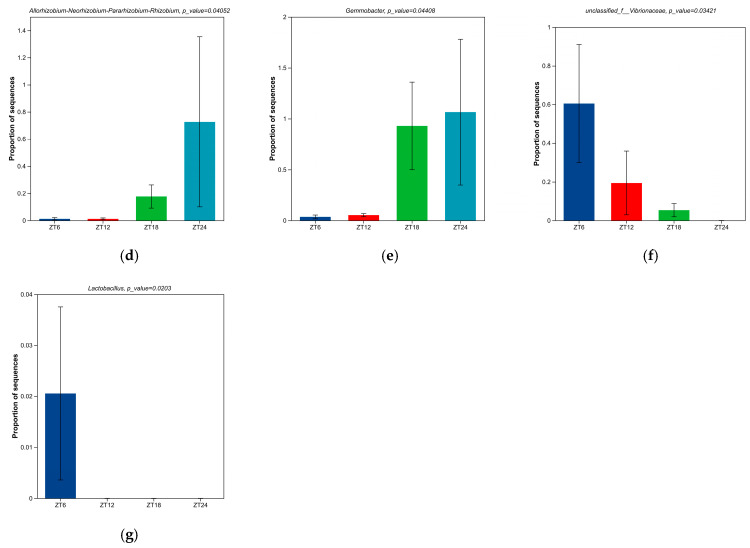
Genera with significantly different relative abundances across sampling time points. (**a**) *unclassified_c_Bacilli*; (**b**) *unclassified_f_Rhodobacteraceae*; (**c**) *unclassified_f_Caulobacteraceae*; (**d**) *Allorhizobium–Neorhizobium–Pararhizobium–Rhizobium*; (**e**) *Gemmobacter*; (**f**) *unclassified_f_Vibrionaceae*; (**g**) *Lactobacillus*.

For the intergroup differences at the genus level, the relative abundances of *unclassified_c_Bacilli*, *unclassified_f_Caulobacteraceae*, and *unclassified_f_Rhodobacteraceae* peaked at 18:00. The abundances of *Allorhizobium–Neorhizobium–Pararhizobium–Rhizobium* and *Gemmobacter* started increasing at 18:00 and reached the peaks at 24:00. In contrast, the abundances of *Lactobacillus* and *unclassified_f_Vibrionaceae* peaked at 06:00 (Figure 6).

### 2.4. mRNA Expression of Core Clock Genes

**Figure 7:**
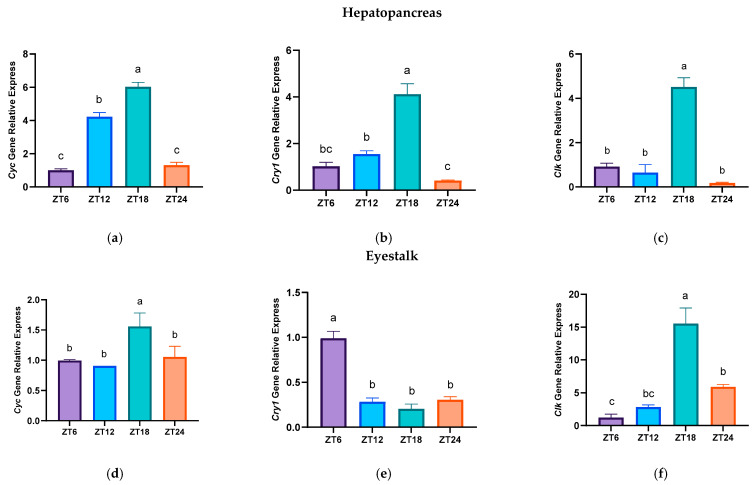
Relative mRNA expression in *Cyc*, *Cry1*, and *Clk* genes in hepatopancreas (**a**–**c**) and eyestalk (**d**–**f**). Different letters indicate significant differences (*p* < 0.05).

The relative mRNA abundances of *Cyc*, *Clk*, and *Cry1* exhibited significant time-dependent fluctuations in both the hepatopancreas and eyestalks (Figure 7). In the eyestalk, both *Cyc* and *Clk* reached their peak expression at 18:00; however, while *Cyc* maintained relatively stable levels at other time points, *Clk* showed the lowest abundance at 06:00. In contrast, *Cry1* expression in the eyestalk peaked at 06:00, with no significant fluctuations observed among the other time points. Within the hepatopancreas, *Cyc* and *Cry1* had low expression levels at 06:00 and 24:00, an upward trend at 12:00, and a maximal peak at 18:00. The *Clk* gene also attained its maximum expression at 18:00, whereas its expression remained consistent at all other sampled time points.

### 2.5. Correlation Analysis Between Gut Microbiota and Clock Genes

**Figure 8:**
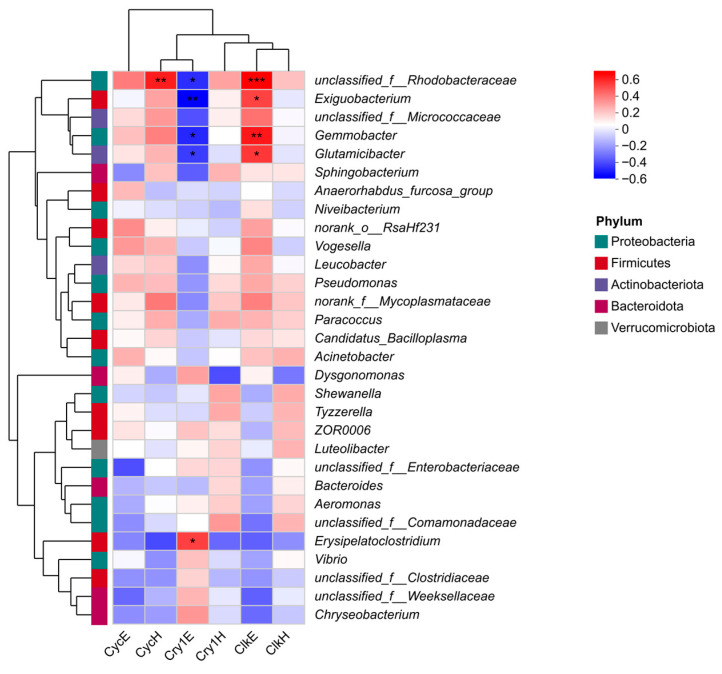
Spearman’s correlation heatmap between 30 genera and 3 clock genes. The color scale on the right shows the color partitioning of the different R values. * *p* < 0.05; ** *p* < 0.01; *** *p* < 0.001. (CycE, *Cyc* gene expression in eyestalk; CycH, *Cyc* gene expression in hepatopancreas; Cry1E, *Cry1* gene expression in eyestalk; Cry1H, *Cry1* gene expression in hepatopancreas; ClkE, *Clk* gene expression in eyestalk; ClkH, *Clk* gene expression in hepatopancreas.).

The correlation analysis between the top 30 bacterial genera and three clock genes is shown in Figure 8. As a result, the abundance of *unclassified_f_Rhodobacteraceae*, *Exiguobacterium*, *Gemmobacter*, and *Glutamicibacter* was significantly negatively correlated with the relative expression level of *Cry1* but significantly positively correlated with the expression level of the *Clk* in the eyestalk. Furthermore, the relative expression level of *Cry1* in the eyestalk was significantly positively correlated with the abundance of the *Erysipelatoclostridium*, and the expression level of *Cyc* in hepatopancreas was significantly positively correlated with the abundance of *unclassified_f_Rhodobacteraceae*.

## 3. Discussion

The gut microbiota plays a pivotal role in maintaining host physiological homeostasis, mediating nutrient metabolism, and modulating the immune system [18,19]. Emerging evidence has highlighted the profound influence of circadian rhythms on gut microbial communities in aquatic crustaceans, such as the *E. sinensis* [7] and *N. denticulata* [9]. Our study provides the first comprehensive characterization of the diurnal variation within the gut microbiota of *P. clarkii*, a species of great global economic value. Although no significant fluctuations were observed in the overall structure across the sampled time points, distinct variation patterns were identified in specific microbial taxa. Meanwhile, diurnal dynamics of core clock gene expression were also observed.

Previous studies in *E. sinensis* [7] and *N. denticulata* [9] have reported significant diurnal oscillations in α-diversity and β-diversity of the gut microbiota. In the current study, however, while α-diversity indices were slightly higher at 18:00, no statistically significant differences were detected among the four sampling time points. This discrepancy may be attributed to species-specific differences and variations in experimental conditions. The culture temperatures in the prior studies were not strictly controlled; for instance, temperature fluctuated between 10 °C and 18 °C in the study of Zhang et al. [9]. In contrast, this study maintained a constant temperature of 20 ± 0.5 °C. Since temperature is one of the key factors affecting the crustacean gut microbiota [20], we propose that the enhanced thermal stability in our study contributed to the relative constancy of the microbial community over the diurnal cycle.

In this study, the predominant phyla in the gut microbiota of the crayfish were Firmicutes, Proteobacteria, Bacteroidota, and Actinobacteriota, which is consistent with previous reports [21,22]. Firmicutes are primarily involved in carbohydrate fermentation and energy metabolism, with their relative abundance often reflecting the host’s nutrient absorption efficiency [23]. Proteobacteria act as both essential nutrient decomposers and opportunistic pathogens, as their significant increase under environmental stress (e.g., cadmium exposure) indicates gut dysbiosis [24]. Bacteroidota are efficient degraders of complex plant polysaccharides, and their abundance is significantly influenced by developmental stage and diet, supporting host utilization of plant-derived nutrients [25]. Actinobacteriota, besides participating in organic matter decomposition, contribute to immune regulation and pathogen suppression by producing diverse secondary metabolites, thereby maintaining gut microecological stability [26].

In some of the other studies, Tenericutes has been reported as a predominant phylum in the gut microbiota of *P. clarkii*, accounting for up to approximately 40% of the total abundance [27,28]. In contrast, Tenericutes was present at a very low abundance in our study. This discrepancy may be partially attributed to the differences in sampling conditions among studies. In the current study, as well as in Xu et al. [21] and Zhang et al. [22], the crayfish were subjected to at least 24 h of starvation prior to gut sampling, whereas no starvation treatment was applied in Liu et al. [27] and Wang et al. [28]. Some Tenericutes species are considered host-associated bacteria that may play roles in nutrient metabolism and digestion [29]. Studies in other crustaceans, including *Litopenaeus vannamei* [30] and *Macrobrachium nipponense* [31], have reported significantly lower abundances of Tenericutes under starvation conditions. Therefore, starvation may partially contribute to the reduced abundance of Tenericutes in this study and similar studies, although other factors, such as culture conditions, diet, and methodological differences, cannot be excluded.

Although no significant differences were observed in α- and β-diversity across the four sampling time points, the relative abundances of several taxa exhibited clear oscillatory patterns. Specifically, at the genus level, *unclassified_c_Bacilli*, *unclassified_f_Caulobacteraceae*, and *unclassified_f_Rhodobacteraceae* all reached their peak abundance at 18:00, coinciding with both the feeding and the onset of darkness. Members of the class Bacilli can secrete various extracellular enzymes that initiate the breakdown of complex dietary substrates, thereby improving host digestive activities [32,33]. Similarly, taxa within Caulobacteraceae are typically associated with the utilization of dissolved organic matter [34], while members of Rhodobacteraceae contribute to central carbohydrate metabolism [35]. Furthermore, *Gemmobacter* and *Allorhizobium–Neorhizobium–Pararhizobium–Rhizobium* showed an upward trend starting from 18:00, attaining maximal levels at 24:00. Gemmobacter has been reported to participate in denitrification processes [36] and is involved in carbohydrate and lipid metabolism [37]. The nitrifying and nitrogen-fixing *Allorhizobium–Neorhizobium–Pararhizobium–Rhizobium* can further enhance nutrient absorption [38,39]. The co-occurrence of these taxa at these time points implies a possible temporally coordinated metabolic process, in which complex dietary components are degraded into simpler substrates.

In contrast, the highest abundances of *Lactobacillus* and *unclassified_f_Vibrionaceae* were at 06:00. *Lactobacillus* is widely recognized for its ability to ferment low-molecular-weight carbohydrates, suggesting its potential involvement in the utilization of simple substrates generated during prior digestive processes. The diurnal oscillation of *Lactobacillus* can be driven by the rhythmic feeding activity [40]. Members of Vibrionaceae are regarded as opportunistic pathogens or transient members in the gut microbiota, and they can bloom in response to substrate pulse [41,42]. The co-occurrence of these taxa at 06:00 reflects a dual ecological strategy, involving both efficient fermentation of available substrates and opportunistic expansion under nutrient-rich conditions. Given that the feeding regimen was synchronized with the light–dark transitions (06:00 and 18:00), these two potent zeitgebers may act in concert to entrain the diurnal dynamics of specific bacterial taxa.

It has been reported that microbial metabolites such as short-chain fatty acids and bile acids can modulate circadian gene expression in peripheral tissues, while host clock genes actively shape the composition and rhythmic dynamics of the gut microbiota [43,44]. For instance, circadian rhythm perturbation directly alters the abundance of Erysipelatoclostridium, and dietary interventions modulate its circadian fluctuations [45,46]. To explore the potential interaction between the observed gut microbiota diurnal dynamics and host molecular regulation, we further examined the circadian expression patterns of core clock genes (*Cyc*, *Clk*, and *Cry1*) in the hepatopancreas and eyestalk of *P. clarkii*. These correlative patterns we observed (Figure 8) suggest that specific bacterial taxa may be functionally linked to the host’s circadian machinery, possibly through the production of short-chain fatty acids (SCFAs) or other microbial metabolites, as shown in other animals [39]. However, given the 24 h fasting condition and the likelihood of host-driven endogenous substrate pulses, these correlations could also reflect a passive response of bacteria to rhythmic host secretions rather than an active, bidirectional crosstalk. Moreover, the tissue-specific correlation profiles (e.g., *Cry1* in eyestalk vs. *Cyc* in hepatopancreas) are intriguing, but their interpretation is constrained by the experimental design.

The mRNA expression levels of *Cyc*, *Clk*, and *Cry1* in the hepatopancreas and eyestalk exhibited time-dependent fluctuations. The expression levels of *Cyc* and *Clk* reached their highest values at 18:00 in both tissues. In contrast, *Cry1* showed tissue-specific rhythmicity, with peak expression at 06:00 in the eyestalk but at 18:00 in the hepatopancreas. Fanjul-Moles et al. reported that the Cry protein levels in the eyestalk peaked at 03:00 [47]. Although differences in experimental design and sampling resolution may account for variation in peak timing, the overall temporal patterns are broadly consistent, with elevated expression occurring during the late night to early morning phase. Notably, the tissue-specific expression pattern of *Cry1* may reflect the distinct physiological roles of these tissues. The eyestalk, as a major neuroendocrine center in crustaceans, is highly responsive to light cues and plays a key role in feeding–light entrained dynamics. In contrast, the hepatopancreas is primarily involved in digestion and metabolism, and its rhythmic gene expression may be more closely associated with feeding-related processes. Overall, the expression of these clock-related genes likely reflects the diurnal behavioral rhythm of *P*. *clarkii*, which is characterized by increased activity, feeding, and metabolic processes during the night, followed by a transition to a relatively stable physiological state during the daytime.

Based on these findings, we propose that optimizing the feeding regimen could enhance the culture efficiency of *P. clarkii*. Given that the peak expression of metabolic-related clock genes in the hepatopancreas and the maximum abundance of digestive-enhancing microbiota both occur at 18:00, providing a single daily meal at the onset of darkness may better align with the host’s natural physiological and metabolic rhythms. Such a strategy could potentially improve nutrient assimilation, reduce feed waste, and minimize water quality deterioration compared to multiple or daytime feeding schedules. Previously, Kardal and Türkmen (2018) compared the effects of feeding frequency on juvenile *P. clarkii*, finding that the survival rate was higher with twice-daily feeding compared to four times daily or every other day [48]. These findings suggest a hypothesis that feeding aligned with peak metabolic activity (18:00) may improve efficiency; however, controlled trials are required.

Several limitations should be acknowledged. First and most critically, the synchronization of the feeding regimen (06:00 and 18:00) with the light–dark transitions (lights on at 06:00) and the coincident sampling times does not allow us to distinguish whether the observed rhythmicity reflects endogenous circadian regulation, direct responses to feeding stimuli, or, most likely, an integration of both. Moreover, the crayfish were starved for 24 h prior to and during sampling, yet they had been entrained to a 12:12 light–dark cycle with scheduled twice-daily feeding for 14 days. Rhythmic feeding schedules are known to elicit anticipatory digestive secretions (e.g., enzymes, mucus, and cell sloughing) even without food [49,50]. Consequently, the observed diurnal peaks in bacterial abundance could partly reflect passive responses to host-derived endogenous substrates rather than autonomous bacterial rhythms. Our design does not allow us to unequivocally distinguish between host-driven substrate pulses and true endogenous rhythms of the gut microbiota. Therefore, in the strictest sense, the patterns we have documented should be described as feeding- and light-entrained dynamics. Second, the six-hour sampling interval in this study may lack the temporal resolution required to capture more rapid or transient fluctuations in specific bacterial taxa. Nevertheless, these findings provide a robust foundation for understanding the diurnal dynamic characteristics of the gut microbiota and core clock gene expression in *P. clarkii*.

## 4. Materials and Methods

### 4.1. Animals

A total of 60 healthy adult crayfish (22.98 ± 3.45 g), with an equal number of males and females, were collected from local markets in Qianjiang, Hubei Province and Jinhu, Jiangsu Province, China, in August 2024. The crayfish were then transported to the aquaculture laboratory in Shenyang Agricultural University.

To mitigate the stress responses in the experimental animals, crayfish were acclimated for 14 days in three dark gray plastic culture tanks (60 × 40 × 23 cm; 20 individuals per tank). The photoperiod was set to 12 h light (06:00 to 18:00) and 12 h darkness, with a light intensity of 1000 ± 50 lx. The water temperature was maintained at 20 ± 0.5 °C. During the acclimation period, the crayfish were fed a commercial compound diet twice daily at 06:00 and 18:00 at a feeding rate of 5% of body weight, allowing ad libitum consumption. In addition, 50% of the tank water was replaced daily. Feeding was suspended 24 h prior to the start of the formal experiment.

Sampling commenced at 06:00 and was conducted every 6 h, resulting in four time points: 06:00, 12:00, 18:00, and 24:00, designated as ZT6, ZT12, ZT18, and ZT24, respectively. Crayfish were anesthetized on ice before dissection. Under aseptic conditions, the entire intestine was excised and placed into sterile 1.5 mL microtubes for subsequent gut microbiota analyses. Eyestalks and hepatopancreases were also collected for gene expression analysis. Six biological replicates (two from each tank) were prepared for each time point, snap-frozen in liquid nitrogen, and stored at −80 °C until further analysis.

### 4.2. Microbiota Sequencing and Data Analyses

#### 4.2.1. DNA Extraction and PCR Amplification

Microbial genomic DNA was extracted from 200 mg of crayfish gut samples using the E.Z.N.A.^®^ Soil DNA Kit (Omega Bio-tek, Norcross, GA, USA) following the manufacturer’s protocol. DNA quality was assessed by 1% (*w*/*v*) agarose gel electrophoresis, and DNA concentration and purity were determined using a NanoDrop™ 2000 UV-Vis spectrophotometer (Thermo Fisher Scientific, Wilmington, DE, USA).

The V3–V4 hypervariable region of the bacterial *16S rRNA* gene was amplified with the primer set 338F (5′-ACTCCTACGGGAGGCAGCAG-3′) and 806R (5′-GGACTACHVGGGTWTCTAAT-3′) on an ABI GeneAmp^®^ 9700 thermal cycler (Applied Biosystems, Foster City, CA, USA). PCRs (20 µL) contained 10 ng template DNA, 4 µL 5 × TransStart FastPfu buffer, 2 µL 2.5 mmol/L dNTPs, 0.8 µL of each primer (5 µmol/L), 0.4 µL TransStart FastPfu DNA Polymerase (TransGen Biotech, Beijing, China), and nuclease-free water to volume. Thermal cycling consisted of initial denaturation at 95 °C for 3 min, followed by 27 cycles of 95 °C for 30 s, 55 °C for 30 s, and 72 °C for 30 s, followed by a final extension at 72 °C for 10 min, and cooling to 4 °C. All reactions were performed in triplicate, pooled, and purified from 2% agarose gels using the AxyPrep DNA Gel Extraction Kit (Axygen Biosciences, Union City, CA, USA) according to the manufacturer’s instructions. Purified amplicons were quantified with a Quantus™ Fluorometer (Promega, Madison, WI, USA) prior to downstream library construction.

#### 4.2.2. Illumina Sequencing

Purified amplicons were pooled in equimolar amounts and subjected to paired-end sequencing on an Illumina Nextseq 2000 platform (Illumina, San Diego, CA, USA) according to standard protocols of Majorbio Bio-Pharm Technology Co., Ltd. (Shanghai, China). The raw reads were deposited into the NCBI Sequence Read Archive (SRA) database (Accession Number: SAMN56704847-SAMN56704870).

#### 4.2.3. Amplicon Sequence Processing

After demultiplexing, sequences were quality-filtered with fastp (v0.20.0) [51] and merged with FLASH (v1.2.7) [52]. High-quality sequences were denoised using the DADA2 [53] plugin in the QIIME2 (version 2024) [54] with recommended parameters, which allows single-nucleotide resolution based on sample-specific error profiles. DADA2 denoised sequences are referred to as amplicon sequence variants (ASVs). To minimize the effects of sequencing depth on α- and β-diversity measures, the number of sequences per sample was rarefied to 40,000, resulting in an average Good’s coverage of 99.97%. Taxonomic assignment of ASVs was performed using the Naive Bayes consensus taxonomy classifier implemented in QIIME2 and the SILVA *16S rRNA* database (v138.2).

#### 4.2.4. Statistical Analyses

Bioinformatic analysis of the gut microbiota was carried out using the Majorbio Cloud platform (https://cloud.majorbio.com, accessed on 15 February 2026) [55]. Based on the ASV information, α-diversity indices, including Shannon, Simpson, ACE, and Chao1, were determined by Mothur 1.30.2 [56]. The similarity among the microbial communities in different samples, β-diversity, was estimated via Bray–Curtis dissimilarity matrices and presented using principal coordinate analysis (PCoA) and non-metric multidimensional scaling (NMDS) using the Vegan v2.5-3 package. The linear discriminant analysis (LDA) effect size (LEfSe) [57] was performed to identify the significantly abundant taxa (from phylum to genera) of bacteria among the different groups, using default parameters (Kruskal–Wallis *p* < 0.05, pairwise Wilcoxon rank-sum test, and LDA score threshold > 2.0). To correct for multiple hypothesis testing, the Kruskal–Wallis *p*-values from all taxonomic comparisons were subsequently adjusted using the Benjamini–Hochberg false discovery rate (FDR) method. Only taxa that satisfied both an FDR-adjusted *p*-value < 0.05 and an LDA score > 2.0 were considered statistically significant biomarkers. Significance was accepted at *p* < 0.05. Results are presented as means ± standard error of the mean (SEM).

### 4.3. Clock Gene Expression

The expression of three core clock genes, including *Cycle* (*Cyc*), *Clock* (*Clk*), and *cryptochrome type 1* (*Cry1*), was assessed through quantitative real-time PCR (qPCR). At each time point, six crayfish were euthanized, and hepatopancreas and eyestalk tissues were immediately dissected on ice for RNA extraction. Gene-specific primers described by Xie et al. [58] were used (Table 1), with the *β-actin* gene serving as the internal reference gene. Reverse transcription and qPCR were carried out in 20 μL reactions using Hiscript^®^ III RT SuperMix for qPCR (+gDNA wiper; Vazyme Biotech, Nanjing, China) following the manufacturer’s recommended cycling parameters. Relative transcript abundance was calculated using the 2^−∆∆Ct^ method to determine fold changes in gene expression.

The statistical analyses were performed using the SPSS 22.0 software package (SPSS Inc., Chicago, IL, USA). Prior to one-way ANOVA, the normality of the data was assessed using Shapiro–Wilk tests, and homogeneity of variances was verified using Levene’s tests for each clock gene in each tissue at each time point. All data met the assumptions for parametric ANOVA (Shapiro–Wilk, *p* > 0.05; Levene’s, *p* > 0.05). Values of the relative mRNA expression of three clock genes (*Cyc*, *Clk*, *Cry1*) were expressed as the mean ± SEM and compared using a one-way analysis of variance (ANOVA), followed by Bonferroni’s multiple comparisons post hoc test. Statistical significance was declared at *p* < 0.05.

### 4.4. Correlation Analysis Between Gut Microbiota and Clock Genes

The Spearman correlation coefficients between top 30 bacterial genera and 6 clock genes were calculated and visualized by the corrplot package in R (3.3.1).

## 5. Conclusions

Our findings demonstrate the diurnal dynamics of the host’s core clock gene expression and specific microbial taxa in *P. clarkii* over a 24 h period. While the overall structure remained relatively stable, the time-dependent fluctuations of functional groups, such as Bacilli and Lactobacillus, may be linked to the host’s feeding activity and light–dark cycles. However, given the 24 h fasting condition, these fluctuations could also reflect passive responses to rhythmic host-derived endogenous secretions rather than autonomous bacterial rhythms. The synchronization between clock gene expression and digestive microbiota at 18:00 provides a potential basis for optimizing feeding strategies. These findings suggest a hypothesis that feeding aligned with peak metabolic activity (18:00) may improve efficiency; however, controlled trials are required. Overall, these insights provide a preliminary scientific framework for developing chronobiological approaches in *P. clarkii* aquaculture, but further studies with uncoupled photoperiod and feeding schedules are needed to confirm the underlying mechanisms.

## Figures and Tables

**Table 1:** Primers for clock gene expression.

Target Gene	Forward (5′–3′)	Reverse (5′–3′)
*β-actin*	GCACCATCCACCATGAAGATTA	CGTGAAAGGGAAGCCAAGATG
*Cyc*	CYGATGTTCCACAGGGACTTAC	GAATCGGCCTCCTCTTTCAA
*Clk*	AAGATGGAGTGCTGGACTTTAG	GCATAAGGGAGCTCTGAAACAA
*Cry* *1*	AGTCAGTGCAACGAGCTGCGAAAT	ACGTGGAGGTGATGGGTATGGTC

## Data Availability

The raw sequencing data of gut microbiota presented in this study are available in the NCBI Sequence Read Archive (SRA) under accession number SAMN56704847-SAMN56704870. The qPCR data supporting the findings of this study are available from the corresponding author upon reasonable request.
